# ‘Do we know if we need to reduce head impact exposure?’: A mixed-methods study highlighting the varied understanding of the long-term risk and consequence of head impact exposure across all stakeholders at the highest level of rugby union

**DOI:** 10.17159/2078-516X/2022/v34i1a13839

**Published:** 2022-01-01

**Authors:** L T Starling, C McKay, M Cross, S Kemp, K A Stokes

**Affiliations:** 1 Centre for Health and Injury and Illness Prevention in Sport, Department for Health, University of Bath, Bath, UK; 2 Premiership Rugby Limited, Twickenham, UK; 3 Rugby Football Union, Twickenham, UK; 4 London School of Hygiene and Tropical Medicine, London, UK

**Keywords:** contact, concussion, head collision, training

## Abstract

**Background:**

One strategy to prevent and manage concussion is to reduce head impacts, both those resulting in concussion and those that do not. Because objective data on the frequency and intensity of head impacts in rugby union (rugby) are sparse, stakeholders resort to individual perceptions to guide contact training. It is unknown whether there is a level of contact training that is protective in preparing elite players for contact during matches.

**Objectives:**

This study aimed to describe how contact training is managed in elite male rugby, and how staff and players perceive contact training load and head impact load.

**Methods:**

This was a sequential explanatory mixed-methods study. Forty-four directors of rugby, defence coaches, medical and strength/conditioning staff and 23 players across all 13 English Premiership Rugby Union clubs and the National senior team participated in semi-structured focus groups and completed two bespoke questionnaires.

**Results:**

The study identified the varied understanding of what constitutes head impact exposure across all stakeholder groups, resulting in different interpretations and a range of management strategies. The findings suggest that elite clubs conduct low levels of contact training; however, participants believe that some exposure is required to prepare players and that efforts to reduce head impact exposure must allow for individualised contact training prescription.

**Conclusion:**

There is a need for objective data, possibly from instrumented mouthguards to identify activities with a high risk for head impact and possible unintended consequences of reduced exposure to these activities. As data on head impact exposure develop, this must be accompanied with knowledge exchange within the rugby community.

Data for match and training time-loss injuries occurring at the top level of men’s rugby in England have routinely been collected since 2002.^[1]^ These data reveal that concussions account for ~25% of all match injuries.^[1,2]^ Most players recover from concussion and return to play without clinical complications in the short-term^[3]^, but it has been suggested that in the medium-to-long term, for some individuals, concussion may contribute to impaired performance, an increased risk of subsequent injury and accelerated neurocognitive decline.^[4]^ Whilst attention has mostly been on the recognition and management of concussions over the last decade, in rugby union the emphasis has more recently turned to primary prevention.^[5–7]^ Rugby’s welfare focus has also expanded from a consideration of concussive events to encompass all significant head impacts. One element of these efforts is to seek ways to reduce unnecessary head impacts. Reducing the incidence of concussions is important; however, it is also important to reduce the frequency of head impacts that do not cause concussions as some evidence suggests that these may also contribute to cognitive decline in the long-term.^[8]^ Repeated sub-concussive impacts have been demonstrated to alter white matter structure and cerebral function.^[9]^ While the clinical significance of these alterations is not fully understood, it has been speculated that this could lead to neurodegenerative conditions, such as chronic traumatic encephalopathy.^[8]^

Little is known about the frequency and magnitude of head impacts in any rugby activity; however, elite players spend the majority (~93%) of their total active training and match play time in training, with ~20% of this training time spent undertaking ‘contact’ activities.^[10]^ Altering the components of match play to reduce head impact exposure is challenging without fundamental law changes but coaching and performance staff have greater control over training activity, and thus training should be considered as a focus for head impact reduction.

Knowledge and perceptions regarding head impact exposure amongst coaching, medical, conditioning and player stakeholder groups in rugby is largely unknown. With limited objective data on head impacts available to guide training prescription, it is likely that these groups’ views influence practice. Therefore, the aims of this study were to describe: (1) how contact training is managed at the elite level of rugby, and (2) how staff and players at this level perceive head impacts and ways to reduce head impact exposure.

## Methods

### Study design

**Fig. 1:**
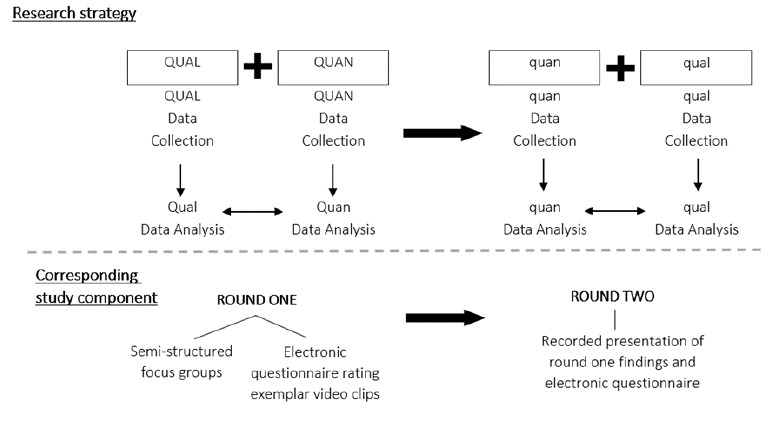
Schematic of sequential explanatory, concurrent triangulation, study design.

This was a pragmatic mixed-methods study conducted from June – August 2021. It followed a sequential explanatory strategy with both primary and explanatory phases of data collection taking the form of concurrent triangulation, whereby quantitative (quan) and qualitative (qual) data were collected at the same time and combined at the point of interpretation (Figure 1). Both qualitative and quantitative approaches were used in this study to find convergence or corroboration and complementarity, using results from one method to elaborate or clarify results from the other.^[11]^

### Participants

Data collection occurred in two remotely conducted rounds with staff and players in all 13 English Premiership rugby clubs (the highest level of club rugby in England) and the National England senior men’s team. The Directors of Rugby, Defence coaches, Heads of Medical Services and Head Strength and Conditioning coaches from each of the 13 English Premiership rugby clubs and the National senior team were purposively targeted for recruitment, as they would be best placed to comment on club and national level approaches to contact training and player management. Staff were contacted directly via email by Premiership Rugby to initiate recruitment. Three players from each of the 13 clubs were also targeted for recruitment as they characterised a range of ages, playing positions, and club and National team representation. Players were contacted directly by the Rugby Players’ Association to initiate recruitment. Individual informed consent was captured via electronic signature from all participants. All staff and players targeted for recruitment agreed to participate in the study. Ethical approval was granted by the Research Ethics Approval Committee for Health at the University of Bath (Ref:20/21 047).

### Procedures

#### Round one

Online meetings were facilitated with each of the 13 Premiership clubs and the National team, with participating staff attending their respective club meetings. Five separate player meetings were held, each comprising a range of club representation. Online meetings began with a 15-minute presentation where current data pertaining to head impact exposure in matches and training in elite rugby were presented. Then, a semi-structured focus group discussion was facilitated by LS. LS has been working in professional rugby research in England for two years and was familiar with some of the participants prior to the focus group discussion but has no rugby playing or coaching experience. A topic guide (Supplementary material 1) was used to guide the focus group process; however, participants were encouraged to say as much as they wished. LS encouraged each participant to provide a response to all questions to ensure the views of the different rugby departments were reflected. The focus groups concentrated on how contact training is defined and managed in the participants’ clubs, their opinions on head impact exposure, and possible opportunities to reduce head impact exposure in training. Focus groups were audio recorded for transcription which lasted between 30–45 minutes. Immediately following each meeting, participants were sent a link to a RedCap questionnaire (Research Electronic Data Capture, Version 8.11.7)^[12]^ (Supplementary material 2). The quantitative questionnaire captured further information on clubs’ contact training scheduling and required participants to rate the potential for head impact exposure, as either *low*, *medium, high*, seen in 15 exemplar video clips of common training activities (e.g. tackle drills with bags, breakdown drills). The questionnaire was assessed for face and content validity through iterative consultation with academic and individuals with specific rugby relevant expertise. This included selecting the video clips in consultation with these experts to ensure the range of all possible training activities was represented.

#### Round two

In Round two, a link to a RedCap questionnaire (Supplementary material 3) was sent to all participants, which included a 15-minute recorded presentation of the findings from Round one, followed by a series of quantitative and qualitative questions to ascertain if participants agreed with the findings. Qualitative questions also probed areas where a lack of convergence emerged in Round one. Data from Round two were analysed and integrated with results from Round one to provide credibility to the findings and substantiate areas where there was a lack of agreement between participants.^[13]^

### Data analysis

Focus group data were analysed using inductive thematic analysis based on the methods suggested by Braun and Clarke^[14]^: transcripts were read several times for familiarisation and inductive semantic coding was used to identify patterns in the data. Higher order themes were developed iteratively following a recursive process of reviewing and defining of emerging concepts, with deduced themes discussed amongst the authors to contribute to the trustworthiness of the analysis.^[11]^ Qualitative analysis was conducted with the assistance of NVivo (QSR International; Version 12 Pro).

Quantitative analysis of questionnaire data was conducted using STATA (StataCorp Version 16.1, 2019). Differences in questionnaire responses between staff and players were assessed descriptively, given the exploratory nature of the research questions. For each video clip, consensus regarding the potential for head impact was established if a category (*low*, *medium*, *high*) was selected by ≥70% of participants.^[15]^

Principles from thematic network analysis were used to develop a graphic of emergent themes and the relationships between them to facilitate understanding in the interpretation of the analysis.^[16]^ Thematic networks graphically present emergent themes as web-like nets to remove any notion of hierarchy, giving fluidity to the themes and emphasising the interconnectivity between them.^[16]^ The relationships between themes were established based on the authors’ interpretation of the data and connectivity between themes. To corroborate the researchers’ interpretation of the findings and give transparency and credibility to the research, participant quotes from the focus groups are provided throughout the results.

## Results and Discussion

Participant demographics are presented in Table 1. The low response rate in Round two is a limitation of the study; however, the representation of all demographic groups and unanimity in responses provide credibility to these responses.

**Fig. 2:**
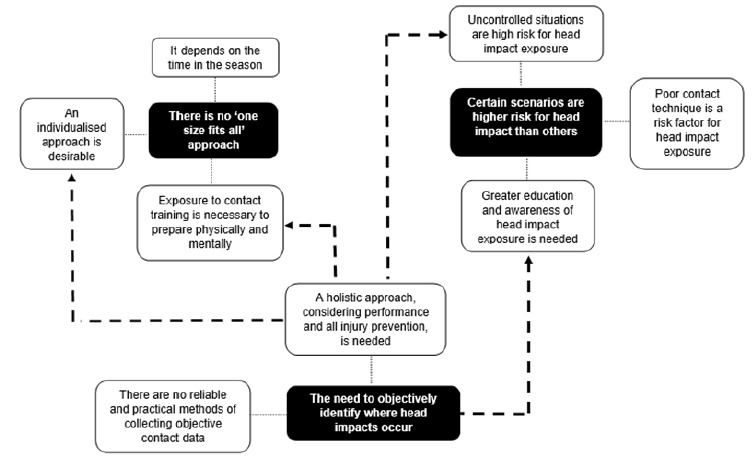
Thematic Network Analysis. Main themes presented in black boxes and sub-themes in white.

From the data, three main themes emerged: **(1) There is no ‘one size fits all’ approach**, with three sub-themes: *1.1: it depends on the time in the season*, *1.2: exposure to contact training is necessary to prepare physically and mentally*, and *1.3: an individualised approach is desirable*. **(2) Certain scenarios are higher risk for head impact than others**, with three sub-themes: *2.1: uncontrolled situations are high risk for head impact exposure*, *2.2: poor contact technique is a risk factor for head impact exposure* and *2.3: greater education and awareness of head impact exposure is needed*. **(3) The need to objectively identify where head impacts occur**, with two sub-themes: *3.1: a holistic approach, considering performance and all injury prevention, is needed* and *3.2: there are no reliable and practical methods of collecting objective contact data*. A thematic network is presented in Figure 2.

### Theme 1: There is no ‘one size fits all’ approach

#### 1.1. It depends on the time in the season

Staff and players indicated that the weekly volume and composition of contact training varied depending on the time in-season. On average, backs were reported to spend eight minutes per week and forwards 23 minutes per week in full-contact training activity. Participants said contact load progressed over the preseason, with greater volumes of full contact training done in a typical preseason week compared to a typical in-season week. This is in line with previous research in professional rugby showing increased exposure to semi- and full-contact training towards the end of preseason, with greater total volumes of training completed in preseason weeks compared to in-season weeks.^[10]^

In-season, the number of days between matches and the stage of the season were taken into consideration. Less contact training was typically done in weeks with a short turnaround between matches and in late-season weeks compared to early-season weeks.

#### 1.2. Exposure to contact training is necessary to prepare physically and mentally

It was unanimous across staff and players that exposure to contact training was necessary to develop and maintain the physical, technical, and mental skills to tolerate the demands of the game, from both performance and injury prevention perspectives. Although there is much debate in the load monitoring literature about appropriate metrics and statistical models, available load monitoring research in rugby shows a U-shaped relationship, with players who are exposed to both low and high training loads being more susceptible to injury than those who are exposed to moderate loads.^[17]^ Participants' views towards contact load appear to reflect this, with participants suggesting that insufficient contact exposure may increase a player's general injury risk if they have not developed the physical and technical ability to overcome the forces associated with contact events. Furthermore, rugby matches were described to be unstructured and unpredictable, and participants highlighted the importance of practising skill execution under these conditions.

*‘If you train a skill from a purely technical point of view you then have to take it to an unstructured point as well, whereby they have to anticipate what’s happening around them, otherwise it’s so structured that they don’t know how to make the correct decision under pressure.’ **Staff 3***

Forwards indicated that scrumming and mauling are integral parts of match events for their position and felt it important for both technical development and body conditioning aspects that they had some exposure to these events every week.

* ‘I'd say from a forwards' point of view, as a pack we would definitely want to have a few live scrums and a few live mauls at least each week.’ **Player 12***

#### 1.3. An individualised approach is desirable

Staff indicated that an individualised approach was taken to prescribing contact training, with the player’s position, match exposure, playing experience and injury status being the primary considerations. Staff indicated that older and more experienced players, players with higher match exposure and those experiencing minor injury complaints would typically do less contact training in a week than their counterparts. The concept of ‘top-ups’ was used to describe how individual players may do additional contact training after a session to work on specific areas of weakness. These ‘top-ups’ may be prescribed by coaches or self-opted by players. A few staff indicated that players’ weekly contact training minutes and match contact event numbers were considered when individualising contact training; however, the majority indicated that a subjective approach, based on player observation, informed this individualisation. Only one club used objective impact data from instrumented mouthguards to inform their contact training prescription.

* ‘In terms of data, no – we prescribe on an individual basis, based on injury history, current injury status’ **Staff 40***

Players agreed that contact training was modified for age, experience, and injury status; however, unlike staff, who indicated that contact training was individualised from the outset, players felt that contact training was standardised and only ‘top-ups’ were individualised.

* ‘…if I’m alright to train then you're just doing the same as what everyone else is, which I think is a funny irony when they're managing the metres [of running] so well, there's no real contact-loading management.’ **Player 4***

* ‘it's pretty standardised week to week, sort of irrespective of game load, or how many minutes you played that weekend or even how many minutes you played that year, etc.’ **Player 7***

### Theme 2: Certain scenarios are higher risk for head impact than others

#### 2.1. Uncontrolled situations are high risk for head impact exposure

Participants indicated that efforts to reduce head impact exposure should be targeted to high-risk areas. They perceived training conducted in live, uncontrolled environments to have a high potential for head impact due to its unpredictable nature and adding a level of control would assist in mitigating the risk.

* ‘…the prevalence of head knocks would be in an uncontrolled situation, where it’s very much like a match day scenario in training, that it is unpredictable and suddenly you get your head in the wrong place and you’ve got a head impact... and it’s thus trying to coach in a controlled manner.’ **Staff 9***

Reducing the number of players, distance and speed of a drill were offered as methods of introducing control to training activities. This reflects research on head injury events in rugby matches, showing the highest propensity for a head injury in high-speed tackles or where more than one tackler is present.^[6]^ Incorporating equipment such as tackling shields or carry bags, which are typically padded and used to dampen the impact forces associated with contact, was also suggested. Some players, however, felt that the incorporation of padded equipment increased the risk if the player holding the equipment feels more protected and thus confident to hit the tackling player with greater force than an unprotected player would.

* ‘The pad adds that element of uncertainty, because there’s one guy that’s live [and] there’s one guy that’s not, and you’re running into the pad, and they might hit you a little bit harder than a guy tackling you would.’ **Player 20***

Both staff and players described contact training to be fluid and during a training session, the intensity and level of control may change either intentionally or unintentionally. Situations were described where a training session may unintentionally increase in intensity because of a few players becoming more competitive, an inevitability due to the nature of the sport. Participants indicated that when the intensity of the drill changes and not all players are aware of it, the mismatch in expected intensity poses a risk to unprepared players. It was felt that it is important in live, uncontrolled training that there is a clear understanding amongst players of the expected level of contact and intensity of the session to ensure they are appropriately primed to avoid high-risk situations under pressure. This contrasts with the discussion in sub-theme 1.2 where participants describe a need for some exposure to unpredictable training to simulate match play. It emphasises the complex nature of balancing contact training with safety.

* ‘…however you define it, it doesn’t always end up like that. So, in the competitive component … you can be having a game of touch, and then somebody gets pissed off, so he tackles them hard.’ **Staff 32***

* ‘When the physicality or intensity level is not clearly defined and people are doing different things, I think you can get yourself in some awkward positions.’ **Player 5***

#### 2.2. Poor contact technique is a risk factor for head impact exposure

Staff and players described poor technique as a risk factor for head impact exposure. Players were regarded to be at an increased risk when executing the tackle with incorrect timing, body height or head position. Research has also identified tackle technical deficiencies as a risk factor for head impacts and head injury.^[18,19]^ This research shows tacklers to be at an increased risk for head impact when executing the tackle with their head on the incorrect side of the ball-carrier and when not shortening their steps before contact. Ball-carriers have been shown to be at an increased risk for head impact when their body is in a ‘straight back’ position pre-contact and when not being explosive at the point of contact. ^[18,19]^

* ‘… a lot of guys get head injuries because technically they’ve not executed what they should have done under a severe level of fatigue.’ **Staff 6***

Participants indicated a need for greater coach education on the importance of technical development for both injury prevention and performance improvement, suggesting that coaches are more likely to adopt injury prevention strategies if they also have a performance benefit. Previous research has identified characteristics of tackles shown to have performance benefits^[20]^, providing support for this suggestion. Ball-carrier explosiveness upon contact is associated with better tackle performance and reduced risk of tackle injury.^[20]^ However, tacklers have a higher chance of winning the contest and a reduced risk of injury when tackling with a straight back and the centre of gravity ahead of the support base and shortening steps into contact.^[20]^

Some participants felt a need for greater education on how best to conduct technical development training, suggesting that these sessions should be conducted in controlled environments, with the focus on the correct execution of the technical skill as opposed to conducting repeated efforts in match-like environments. Many players described training sessions aimed at improving an area of weakness in the team to involve conducting repeated efforts of the specific event in match-like conditions. Players felt it would be safer to focus the session on technical execution in a controlled environment. This further highlights the notion in sub-theme 2.1 that conducting training in controlled environments is likely to reduce the risk for head impact exposure.

* ‘Did we miss any tackles at the weekend? Yeah, we missed quite a lot – right, let’s do some live tackling... it’s almost a way of trying to solve a problem, rather than understanding why it went wrong’ **Player 3***

#### 2.3. Greater education and awareness of head impact exposure is needed

All participants described head impact exposure occurring when there was a direct impact to the head, either against another player or the ground. Fewer participants described indirect mechanisms, such as an impact on another body part resulting in a whiplash-type motion of the head. Emerging research making use of instrumented mouthguards (iMGs) to quantify head acceleration events provides evidence for indirect mechanisms, with accelerations to the head being recorded as a result of impact to the body and subsequent momentum transferred to the head.^[21]^ Indirect mechanisms have been reported as the most common cause of head impact in male university level rugby players, (31% of all head impact events), with an uncontrolled whiplash action present in many cases, with this present in ~50% of all female rugby impact events.^[21]^

* ‘I was going to ask you that question because is it just contact with the head or, that whiplash, that sort of a head rattle that people get... I wouldn't know actually, is that part of it?’ **Staff 12***

It was clear that training drills were designed to mitigate against injury to ensure maximum player availability for match team selection, with head impact exposure no more of a consideration than the protection of any other body region.

* ‘Head injuries have an impact, but also other injuries have an impact. If we’re losing lads in training then they aren’t available then the weekend and then whether it’s a head impact, whether it’s a shoulder, we’re doing something wrong.’ **Staff 21***

**Fig. 3:**
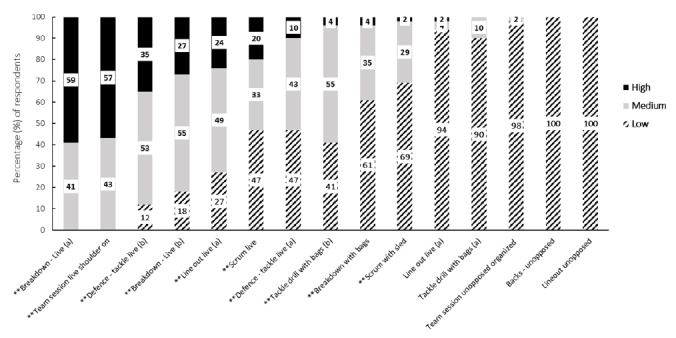
Proportion of respondents rating the potential for head impact exposure as high, medium, or low for training video clips. Data presented as grouped staff and player responses. ** indicates consensus (≥70%) was not reached.

**Fig. 4:**
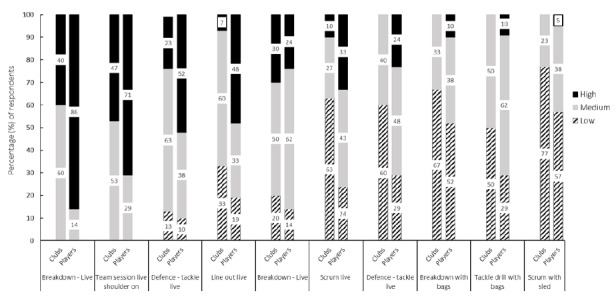
Proportion of respondents rating the potential for head impact exposure as high, medium, or low for training video clips where consensus (≥70%) was not reached. Data presented as staff and player responses separately.

Overall, there was a lack of agreement on the potential for head impact in the 15 training video clips provided in Round one, with 67% (n = 10) of the clips not reaching consensus (Figure 3). Separating the staff and player responses for the clips where consensus was not reached revealed a mismatch in perceived potential for head impact exposure between staff and players. In each of these clips, players rated the potential for head impact exposure to be higher than staff did (Figure 4).

The mismatch between interpretations of the potential for head impact exposure was further explored in the data exploration component of Round two. Both staff and players indicated that this was likely due to the detachment of staff from head impacts in comparison to players experiencing them.

* ‘Players have felt it and many staff only watched it! Much of the potential for head impact is unclear - hence the need for objectivity and more education to remove the ambiguity.’ **Staff 3***

There is a need for greater education on what constitutes a head impact exposure and the magnitude and frequency of head impact exposures in different match and training activities. Enhanced education will equip staff with knowledge on how best to manage exposures in training and will also facilitate alignment between staff and players.

### Theme 3: The need to objectively identify where head impacts occur’

#### 3.1. A holistic approach, considering performance and all injury prevention, is needed

It was unanimous that participants wanted to objectively identify where head impact exposures occur in matches and training. Participants had previously highlighted that efforts aimed at reducing head impact exposure should directly target high-risk areas (Sub-theme 2.1) and they emphasised the need for these to be objectively identified.

* ‘We need to collect more data for a better understanding because then hopefully we can identify drills that will allow us to practice, because we have to practice, but that have minimal exposure to head impacts or high head impacts.’ **Staff 14***

Participants highlighted that exposure to rugby-specific contact activity is necessary (Sub-theme 1.2) and thus expressed the importance of considering possible unintended performance and injury prevention consequences of reduced contact training exposure.

* ‘We’d absolutely support any initiative which would maintain the integrity of the game, by reducing head injuries. I don’t necessarily think that it’s going to be a direct correlation between reducing training contact and reducing head injuries, it might have the inverse effect.’ **Staff 32***

* ‘We could always limit the potential for head knock exposures at training, but on the flip side, you want to train the skill so that you go confident into the weekend. You don’t want to go for a couple of weeks without training it.’ **Player 9***

Staff and players highlighted that increased awareness of player safety over the years has led to a decrease in the overall exposure, and increased level of control, of contact training sessions, leaving them feeling that they are already doing the minimal amount of contact training necessary (Sub-theme 1.2). This opinion contradicts the findings of previous research in the same population, which reports no significant changes in contact training time over an 11-season period.^[10]^

* ‘I don't know how much more we could reduce it be able to go into the weekend confident.’ **Player 10***

In recent years, some contact sports have seen limits placed on the volume and frequency of contact training permitted in a season by their governing bodies [e.g. National Football League (NFL), Canadian Football League (CFL)]. When the possibility of implementing similar in-season constraints was explored with participants in this study, they were conceptually supportive, provided the limits still enabled the necessary contact exposure for appropriate development (Sub-theme 1.2) and an individualised approach (Sub-theme 1.3).

Currently, the national governing body of clubs participating in this study enforces a mandatory five-week no contact training post-season period. When participants were asked about extending the length of that period, several concerns were raised. Participants highlighted that a lack of exposure to contact for an extended time may result in deconditioning and subsequently an increased risk for injury (Sub-theme 1.2). In professional rugby, a greater frequency and burden of training injuries is observed in the early period of the preseason, immediately following the 5-week off-season.^[22]^ The outbreak of the Coronavirus pandemic resulted in disruptions to the 2019–20 English Premiership rugby season and players were subjected to 12-weeks of restricted training.^[23]^ When players returned to team training, a significantly higher incidence of training injury was observed in comparison to that following a regular 5-week off-season.^[23]^ Although players would typically have access to more training facilities during a regular off-season than they did during this 12-week period, the findings provide some support to the participants’ concerns that an extended period of not training specific skills, namely, those that require interaction with other players, may result in deconditioning and a subsequent increased risk for injury.^[23]^ It is likely an individualised approach to training and recovery is required.

* ‘It’s rest, but it’s also detraining. You’ve got to be careful of making that too long, because doing some progressive contact is a form of injury prevention, and coaching technique is a form of injury prevention.’ **Staff 4***

Participants also stressed that developing contact conditioning safely requires a progressive build up and if insufficient time was afforded before the first match of the season, a rapid increase in contact exposure would pose a risk to players (Sub-theme 1.1). In professional rugby, rapid increases and large changes in week-to-week training load have been associated with increased injury risk ^[17]^ and while this applies to the total load and not the contact load specifically, the principles may be the same. The increased risk of injury observed following the Coronavirus-induced suspension of the season^[23]^ appeared to be mitigated by the time competition resumed, with the match injury incidence comparable to the regular season.^[23]^ A 10-week progressed training period was implemented before match play resumed and suggests that an appropriate and progressive return to training may assist in mitigating an increased risk of injury associated with an extended time away from regular match play and team training.^[23]^

* ‘If you're going to put a minimum amount of time you've got to have off then you probably need to think about a maximum amount of time before the contact and how you bridge to that intensity’ **Staff 9***

#### 3.2. There are no reliable and practical methods of collecting objective contact data

The feasibility of collecting objective contact data was discussed in the staff focus group sessions. Several barriers to the collection of objective contact data were identified by staff participants. A few clubs indicated that they have been trialling iMGs, but this data is yet to inform practise sessions due to a lack of understanding and/or confidence in the metrics they produce.

* ‘We've looked at different parameters and talked to different S&C coaches around the clubs and there isn't anything tangible that I would put my confidence in to then talk to the coaches about.’ **Staff 19***

Human resources were also identified as a barrier, with the collection of head impact exposure-related data considered an additional time burden on already busy staff. It is important to note that the only club using data from iMGs to inform contact training prescription had a sports scientist directly linked to the mouthguard provider embedded within the club. This individual was responsible for managing the collection and analysis of iMG data and, having a significant understanding of the metrics produced, was able to work alongside coaching staff to translate the data to practically relevant changes to the contact training schedule.

* ‘…it's something that would be good but the burden at the moment to collect all that information and I also don't think [we] have microtechnology that actually quantifies it correctly.’ **Staff 7***

## Conclusion

This study has identified a varied understanding of what constitutes head impact exposure and the activities that contribute to the greatest magnitude and frequency of exposure across a diverse range of stakeholders in elite rugby. The absence of available research on head impact exposure was a catalyst to conducting this study and the findings confirm that there is a real knowledge gap in the rugby community. A strength of this study is its diversity of participants, exposing that varied knowledge is apparent across medical, conditioning, coaching, player, and research departments at the highest level of rugby. Subjectivity will always be present in coaching, load prescription and player management; therefore, exploring subjective views on the topic is essential. The sequential mixed-methods design of the present study was a strength-based approach, with triangulation of data producing substantiated findings and participant feedback providing credibility to the research outputs.

The absence of objective data to inform understanding of head impact exposure has resulted in different opinions and consequently varied management strategies have emerged. Most notable were the different views on specific elements relating to head impact exposure amongst players compared to staff. Players typically interpreted training activities to have a higher potential for head impact than staff and, while staff suggested the incorporation of padded equipment would make training safer, players viewed this as potentially increasing the level of risk. There were also mixed interpretations of how best to conduct training for technical development, while limiting head impact exposure, with some participants indicating these sessions are best achieved through training in controlled environments and others indicating that repeated efforts in match-like environments are necessary. Yet, participants felt that they have already limited the amount of full contact training to the perceived minimum necessary amount, and that no further decrease is possible. These findings show that individuals within clubs have different interpretations of head impact exposure, resulting in sometimes contradicting management techniques.

To promote open communication, player focus groups were held separately from staff sessions. Nonetheless, it is probable that the format of having all staff from each club present during one online session may have made it harder for some staff to communicate freely than if sessions had been guided 1-2-1 or in-person. Coronavirus-induced constraints on conducting in-person research, combined with time constraints on participants, with this study being conducted during the final rounds of the season, meant that the options of formats for conducting focus group sessions were limited. These limits may partly explain the low response rate seen in Round 2 of the study. It is difficult to implement change and inform practice when individuals have varied perceptions about a subject and this becomes a greater challenge when there is limited objective data available to support any changes. There is a need for objective data to identify activities with a high risk for head impact exposure and possible unintended consequences of reduced exposure to these activities. The Rugby Football Union, the governing body of the teams involved in this study, has committed itself to make instrumented mouthguards available to all players competing in the upcoming English Premiership season as a result of this work, in order to gather objective impact data and advance knowledge on this subject. As data and knowledge on head impact exposure develop, this must be accompanied with knowledge exchange within the rugby community. Even with objective data available, training prescription will always contain an element of subjectivity and as such, knowledge exchange amongst practitioners will be essential to develop safe but effective training practices. Rugby union is a complex landscape with a diverse range of stakeholders involved in policy development and practise, thus research needs to be combined with expertise and experience from all stakeholder groups to develop practical solutions to generate change in this area.

## Supplementary Information



## Figures and Tables

**Table 1:** Participant demographics

	Round one		Round two

	Qualitative focus groups	Quantitative questionnaire	Mixed-methods questionnaire
**Staff**	**44**	**30**	**12**
Directors of Rugby (DoR)	11	6	3
Coaches in addition to DoR coaches	7	6	2
Head of Medical	12	8	6
Head of Conditioning	14	10	1
**Players**	**23**	**23**	**7**
Backs	10	10	3
Forwards	13	13	4

**Total participants**	**67**	**53**	**19**
