# Supplementary material for: ‘Do we know if we need to reduce head impact exposure?’: A mixed-methods study highlighting the varied understanding of the long-term risk and consequence of head impact exposure across all stakeholders at the highest level of rugby union
Source: S Afr J Sports Med. 2022 Jan 1;34(1):v34i1a13839. doi: 10.17159/2078-516X/2022/v34i1a13839 (PMC9924567; doi:10.17159/2078-516X/2022/v34i1a13839)
Supplement: Supplementary file 1 [file 2078-516X-34-v34i1a13839-s001.pdf]

‘Do we know if we need to reduce head impact exposure?’: A mixed-method study highlighting the varied understanding of the long-term risk and consequence of head impact exposure across all stakeholders at the highest level of rugby union

Questions addressed in manuscript indicated with ✓

Supplementary file 1: INTERVIEW TOPIC GUIDE

		EXPLORE
	CONTACT TRAINING	
	How would you define the different categories of contact training (full-contact, semi-contact, non-contact)?	- Defining a session if pads were used.<br>- Defining a session if suits were used.<br>- Defining a session if conducted at different intensities.
✓	What contact training do you view as being “essential”? With “Essential” pertaining to the development of technical proficiency, to appropriately prepare players for the physical demands of game and to appropriately prepare players for performance expectations.	- Influence of the time in season.<br>- Influence of player position.<br>- Influence of player experience.
✓	Are you currently doing any monitoring of players (i.e., collecting data from players) that informs what contact training you do?	- Experience with iMGs
	HEAD IMPACT EXPOSURE	
✓	When we refer to Head Impact Exposure what do you interpret this to mean exactly?	- Explore consideration of indirect impacts if not mentioned by participants.
✓	With that interpretation in mind, when planning contact training sessions, is head impact exposure a consideration when selecting the activities to include in the session?	
	OPPORTUNITIES TO REDUCE HEAD IMPACT EXPOSURE	
✓	Having reflected on contact training and head impact exposure from a training perspective, and what you feel is essential to meet the technical, physical and performance demands of the game, how to you think we might go about reducing head impact exposure in training?	
✓	What are your thoughts on the concept of a no contact training window at the end of a season? If a window of time was implemented where no contact done would	- Is current 5-week sufficient?<br>- How would lengthening this influence you?
✓	What do you think of the concept of confining all contact training to be on one day of the week?	- Influence of time of season on this.

Supplementary file 2: ROUND ONE QUESTIONNAIRE

✓ **Participant Demographics**

Please indicate your primary job role	◯ Director of Rugby<br>◯ Medical staff member<br>◯ Conditioning staff<br>◯ Rugby Coach<br>◯ Player
Are you a forward or a back?	◯ Forward ◯ Back
In addition to your role as Director of Rugby, do you also fulfil a role of defence or forwards coach at your club?	◯ Yes<br>◯ No

✓ **Current Contact Training Scheduling**

To what extent do you feel the following factors are considered when scheduling contact training sessions in a week?

	Very little extent	Little extent	Neutral	Large extent	Very large extent
Time in season	☐	☐	☐	☐	☐
If there is a match scheduled on the upcoming weekend	☐	☐	☐	☐	☐
The importance placed on the upcoming weekends' match	☐	☐	☐	☐	☐

Are there any additional factors to those above that are taken into consideration?	◯ Yes<br>◯ No
Please specify these factors	____

Pre-season Contact Training for Backs

During a typical week in the pre-season period: How much contact training (in minutes) is scheduled in the week for backs ?

During a typical week in the pre-season period:
On what days, and time of day, would contact training typically be scheduled for backs ?
Please select all that apply

	Monday	Tuesday	Wednesday	Thursday	Friday	Saturday	Sunday
Early Morning	☐	☐	☐	☐	☐	☐	☐
Late Morning	☐	☐	☐	☐	☐	☐	☐
Early Afternoon	☐	☐	☐	☐	☐	☐	☐
Late Afternoon	☐	☐	☐	☐	☐	☐	☐

✓

During a typical pre-season week, for backs, does contact training get conducted as a component of a predominantly non-contact session; or does it get conducted as a dedicated contact only session?	◯ a) Component of a non-contact session<br>◯ b) Dedicated contact only session<br>◯ c) Mixture of both a) & b)

Pre-season questions repeated for Forwards

In-season Contact Training for Backs

The questions below apply to an in-season week leading up to a match with regular turn around time for backs

In a regular in-season match week : How much contact training (in minutes) is typically scheduled in the week for backs ?

During a typical in-season match week:
On what days, and time of day, would contact training typically be scheduled for backs ?
Please select all that apply. MD= Match Day.

	MD-6	MD-5	MD-4	MD-3	MD-2	MD-1	Match Day (MD)
Early Morning	☐	☐	☐	☐	☐	☐	☐
Late Morning	☐	☐	☐	☐	☐	☐	☐
Early Afternoon	☐	☐	☐	☐	☐	☐	☐
Late Afternoon	☐	☐	☐	☐	☐	☐	☐

✓ During a typical in-season week, for backs, does contact training get conducted as a component of a predominantly non-contact session; or does it get conducted as a dedicated contact only session?

◯ a) Component of a non-contact session
◯ b) Dedicated contact only session
◯ c) Mixture of both a) & b)

In-season questions repeated for Forwards

You will now be taken through 15 video clips of training drills (5min duration total). For each video clip please click on the link, which will download the video for you to watch.

After watching the video you will be asked to 1) categorize the level of contact of the training drill and 2) rate the potential for head impact exposure in the training drill. Optional comment boxes are provided for you to substantiate the ratings you give each clip.

"Head Impact Exposure" = Accelerations to the head as a result of forces sustained to the body or head"

CLIP 1
Please click on the link to download and watch the clip.

[Attachment: "Clip 1..mov"]

1.1a How would you categorize Clip 1?

◯ Full-contact
◯ Semi-contact
◯ Non-contact

1.1b Please provide a brief comment to describe why you selected this category.

____

✓ 1.2a What do you think the potential exposure to head impact is in Clip 1?

◯ Low
◯ Medium
◯ High

✓ 1.2b Please provide a brief comment to describe why you selected this category.

____

Questions repeated for each of the 15 Clips described below.

Clip	Description
1	Breakdown - Live
2	Defence - Tackle live
3	Lineout - Live
4	Scrum - Live
5	Backs - Unopposed
6	Team session - Live, Shoulder-on
7	Tackle drill with bags
8	Lineout - Live
9	Breakdown with bags
10	Defence - Tackle live
11	Scrum with sled
12	Lineout - Unopposed
13	Tackle drill with bags
14	Breakdown - Live
15	Team session - Unopposed, Organized

SUPPLEMENTARY FILE 3: ROUND TWO QUESTIONNAIRE

✓

Participant Demographics

Please indicate your primary job role

◯ Director of Rugby
◯ Medical staff member
◯ Conditioning staff
◯ Rugby Coach
◯ Player

Are you a forward or a back?

◯ Forward
◯ Back

In addition to your role as Director of Rugby, do you also fulfil a role of defence or forwards coach at your club?

◯ Yes
◯ No

Proposed Contact Training Definitions

Category	Key Features	Use of suits	Use of shield/bags/ crash mats	Scrum	Maul
Full contact	Live<br>Bone-on-Bone/Suits<br>Uncontrolled	✓ Possible	✗ Never	Live<br>Bone-on-Bone	Live<br>Bone-on-Bone
'Live' semi-contact	MODIFIED FULL CONTACT TRAINING<br><br>Bone-on-Bone/Suits<br>Controlled environment.<br>Reduced speed and/or intensity.<br><br>*"Shoulder on"*<br>*"Healthy Grab"*<br>*"Body in front"*	✓ Possible	✗ Never	Live<br>Bone-on-Bone/Suits<br>Controlled/modified conditions	Live<br>Bone-on-Bone/Suits<br>Controlled/modified conditions
Semi-contact	ALL OTHER FORMS OF CONTACT TRAINING<br><br>Highly controlled, structured environment.<br>Further reductions in speed and/or intensity.<br><br>Use of pads, shields, bags, crash mats.	✓ Possible	✓ Possible	Scrum against machine<br>Scrum focusing on set up	Maul focusing on set-up
Non-contact	UNOPPOSED TRAINING<br><br>No level of intensity or force in contact/collisions between players	✓ Possible	✓ Possible	✗ Never	✗ Never

Do you feel that the proposed contact training definitions described in the presentation and summarized above are a workable set of definitions?

◯ Yes
◯ No

What do you feel would be more appropriate contact training definitions?

____

Are there any comments you would like to provide on these proposed definitions?

____

Based on the newly proposed contact training definitions, in a typical in-season week, how much time (in minutes) do you feel backs spend in full contact training?

Question asked for '*full contact*', '*controlled contact*', and '*semi-contact*', for backs and forwards separately.

✓ 1a If there was a recommendation that for the first two weeks of the 2022-23 pre-season (once players have returned from their mandatory five week break) no "Full contact" and no "'Live' semi-contact" training could be undertaken, would you be supportive of this recommendation?

◯ Yes
◯ No

✓ 1b How would this impact on your pre-season preparation?

____

✓ 2 Thinking about the presentation you have just watched, can you think of any reason why there is a mismatch between players and staff in terms of the interpretation of potential for Head Impact Exposure in the video clips circulated in the survey round?

____

✓ 3 Based on the summary findings presented, and since we last spoke, do you have any further thoughts on potential ways to reduce Head Impact Exposure in training?

____

4a Do you feel that this process has been clear and transparent activity and that it has provided you with sufficient opportunity to input and share your views?

◯ Yes
◯ No

4b Do you have any feedback you would like to provide on how you have found this process to be?

____

You will now been taken through 8 video clips of training drills (3mins duration total) where we would like you to categorize the level of contact of the clip according to the newly proposed contact training definitions.

CLIP 1
Please click on the link to download and watch the clip.

[Attachment: "Clip 1..mov"]

How would you categorize Clip 1?

◯ Full contact
◯ 'Live' Semi-contact
◯ Semi-contact
◯ Non-contact

Please provide a brief comment to describe why you selected this category.

____

Questions repeated for each of the 8 Clips described below.

Clip	Description
1	Breakdown - Live
2	Defence - Tackle live
3	Scrum - Live
4	Team session - Live, Shoulder-on
5	Tackle drill with bags
6	Lineout - Live
7	Tackle drill with bags
	Breakdown – Live
